# Study on Spontaneous Reactivation and Aging of Acetylcholinesterase Inhibited by Paraoxon and Malaoxon in Ten Species

**DOI:** 10.3390/ijms241814213

**Published:** 2023-09-18

**Authors:** Mingwei Gao, Zhongwen Ni, Guo Li, Gang Wu, Binbin Huang

**Affiliations:** Key Laboratory of Biopesticide and Chemical Biology, Ministry of Education, Fujian Agriculture and Forestry University, Fuzhou 350002, China; 1220215001@fafu.edu.cn (M.G.); lgzy918@163.com (G.L.)

**Keywords:** acetylcholinesterase, organophosphate, aging, spontaneous reactivation

## Abstract

Organophosphorus insecticides (OPs), acting as serine phosphorylating agents in acetylcholinesterase (AChE), are highly effective neurotoxic insecticides. In our previous research, we found that six herbivorous pests and four ladybirds howed significantly higher AChE LC50 values than seven parasitoids and a predator (*Epistrophe balteate*), and that there was a significant correlation with the corresponding bimolecular rate constant (Ki) value. The Ki value of pests was much smaller than that of natural enemies and had a higher LC50 value.Then, we speculated that the low sensitivity of the pest AChE to OPs may be associated with its higher recovery and lower aging ability. In this work, the I50 and I90 were calculated, to determine the sensibility of AChE in ten representative species, including *Plutella xylostella*, *Prodenia litura*, *Musca domestica*, and *Cavia porcellus*, to paraoxon and malaoxon. The enzyme activities were measured at various time points, and kinetic calculations were used to obtain their spontaneous reactivation (Ks) and aging (Ka) constants, which were comprehensively compared. We conclude that the Ka and Ks of the AChE inhibited by OPs showed primarily species-specific correlations, and little correlation with the sensitivity to OPs. The differences in the AChE sensitivity to paraoxon among the ten species were much greater than in the sensitivity to malaoxon. Compared to paraoxon, malaoxon was more selective for *Cavia porcellus*. Coleoptera insects showed a stronger dephosphorylation ability than other insect groups. The recovery ability of phospho-AChE was stronger in mammals than in insects, which could be related to the low sensitivity of the AChE site of action to OPs. The Ka of the AChE inhibited by malaoxon was larger than that inhibited by paraoxon with the corresponding biomaterials, indicating that the OP type had a substantial relationship with the Ka of the AChE. We further discovered that, when insects were inhibited by OP, the tendency of AChE to undergo aging was greater than that of dephosphorylation. Overall, the study provides valuable information on the action mechanism of various OPs on AChE in several species, which could be used to further research into AChE and the potential dangers that organophosphates pose to animals.

## 1. Introduction

Acetylcholinesterase (AChE; EC 3.1.1.7) is an important in vivo target for organophosphate (OP) and carbamate pesticides [1]. AChE is found in brain synapses and neuromuscular junctions (NMJs), with a catalytic triad of Glu-His-Ser, which can silence nerve pulses by selectively hydrolyzing acetylcholine, a neurotransmitter [2]. OPs can inactivate AChE by phosphorylating it, which prevents the AChE from hydrolyzing substrates, thereby disrupting the transfer of neurotransmitters, and endangering the lives of animals that come into contact with these pesticides [3].

**Figure 1:**
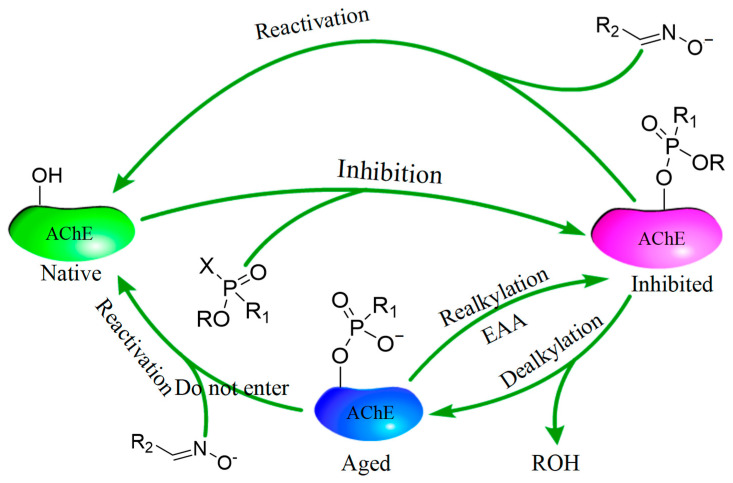
The mechanism of the interaction between organophosphorus agents and AChE (spontaneous reactivation and aging). Green: natural AChE with enzymatic activity; red: AChE induced by an organophosphorus agent; blue: aged AChE. X stands for the group lost during inhibition. EAA, electrophilic alkylating agents. Realkylation may bring the aged AChE back to the inhibited, but not aged, state, and, hence, facilitate reactivation by oximes.

OP compounds are among the most widely used insecticides worldwide [4,5]. OPs such as parathion and malathion can undergo biotransformation via cytochrome P450 enzymes to acquire more toxic forms [6,7]. During the Green Revolution in the 1960s, these insecticides were introduced globally into developing rural agricultural communities who were not adequately prepared to use and store them [8]. According to conservative estimates, more than 100,000 people die yearly from pesticide self-poisoning worldwide, accounting for 13.7% of the global suicide toll [9]. The main mechanism of action of OPs is a progressive inhibition of AChE via the phosphylation (i.e., phosphorylation and phosphonylation) of serine at the active site, leading to enzyme inactivation [10,11]. The failure of AChE to hydrolyze the neurotransmitter acetylcholine results in endogenous acetylcholine intoxication, followed by the over-stimulation of cholinergic receptors, the massive disturbance of numerous body functions, and finally, death from respiratory failure [12,13]. However, the AChE inhibited by OPs can be reactivated by oximes (e.g., 2-PAM) in certain conditions [14] (Figure 1), and anticholinergic drugs (e.g., atropine) can be used for detoxification. When the pesticide is removed from the active site, phospho-AChE will spontaneously dephosphorylate to restore a certain activity level. Phospho-AChE can also undergo dealkylation (aging), making reactivation impossible. Both reactions depend strongly on the OP structure [15].

The inhibiting impact of an OP on AChE varies among species. Similarly, the mechanisms through which the AChE of a species is inhibited vary among insecticides [16,17,18,19]. This means that the research on AChE aging and recovery is of significant importance to the clinical treatment of OP poisoning and insect resistance development in the pest control research. In our previous research, we found that parasitoids, predators, and herbivores exhibited various OP tolerance models and enzymatic potentials based on the insecticide selection pressure (through direct exposure to the spray in the wild or through indirect penetration of the insecticides into the host insect body), and the ecological and biological habitat. Six herbivorous pests and four ladybirdsshowed significantly higher AChE LC50 values than seven parasitoids and a predator (*Epistrophe balteate*), significantly correlating with the corresponding bimolecular rate constant (Ki) value [20]. The Ki value of pests was much smaller than that of natural enemies and had a higher LC50 value. Based on our previous results, and after in-depth discussions, we wondered whether the AChE self-recovery ability was specifically related to the lower sensitivity to OP exhibited by pests than their natural enemies, or whether it was consistent with our other discovery that carboxylesterase (CarE) and glutathione-S-transferase (GST) activities varied among insect species (species-specific) [20]. Enzyme kinetics can clearly reveal the enzyme reaction velocity, and the effect of various factors on the reaction, providing insights into the reaction mechanism, which is crucial to developing safe insecticides with a high selectivity, and to studying the AChE mechanism.

In recent years, domestic and foreign studies have concentrated on the aging process and spontaneous reactivation of AChE in mammalians (e.g., mice, human blood, rabbits, zebrafish, etc.) inhibited by OPs [21,22,23]. Several important achievements with a significant reference value have been reported in these studies. However, the reports on insect-related research are relatively scarce, and none have been published comparing insect species for differences related to AChE. In this study, ten different species, including representative pests, natural enemies, *Cavia porcellus*, *Rana catesbeiana*, and *Carassius auratus*, were used as experimental animals, to systematically compare the aging and reactivation constants of AChE inhibited by paraoxon or malaoxon between pests and their natural enemies, and among species. We believe that our results will be of substantial value to the research on pest resistance mechanisms, species evolution, and parameters associated with AChE.

## 2. Results

### 2.1. Sensitivity of AChE to Paraoxon and Malaoxon

Through toxicity determination, we calculated the I50 and I90 of AChE inhibited by paraoxon and malaoxon in ten different species. The I50 was mainly used to judge the sensitivity of AChE to OPs. We used the I90 concentration drugs to inhibit the AChE samples, ensuring that the initial activity of all the enzyme samples remained at the same level [24]. Table 1 summarizes the sequence of AChE I50 values of the various species inhibited by paraoxons (*C. plutellae* < *P. puparum* < *C. porcellus* < *P. japonica* < *P. striolata* < *P. litura* < *M. domestica* < *P. xylostella* < *C. auratus* < *R. catesbeiana*) and malaoxons (*P. puparum* < *C. plutellae* < *P. japonica* < *P. litura* < *P. striolata* < *M. domestica* < *C. porcellus* < *C. auratus* < *P. xylostella* < *R. catesbeiana*).

As can be seen from the AChE I50 values of the ten different species inhibited by paraoxon and malaoxon, their sensitivity to paraoxon varied considerably, ranging from *R. catesbeiana* as the least sensitive, to *C. plutellae* as the most sensitive, with the former being 2417 times less sensitive than the latter. However, the differences in AChE sensitivity to malaoxon among these biomaterials were significantly smaller than those to paraoxon. The least sensitive AChE was from *R. catesbeiana*, and the most sensitive was from *P. puparum*, with the former being nearly 102 times less sensitive than the latter. The *R. catesbeiana* AChE maintained a much higher insensitivity level to both OPs than the other species. Furthermore, except for the AChE of *C. porcellus*, a comprehensive analysis of the I50 revealed that the AChE of common species sensitive to paraoxon was also sensitive to malaoxon. However, the AChE of *C. porcellus* was very sensitive to paraoxon, but insensitive to malaoxon. It was deduced that malathion was more selective than parathion [25]. The AChE of *P. xylostella* exhibited a much higher IC50 under the inhibition of the two OPs than that of its natural enemy *C. plutellae*, indicating that the heavy use of OP pesticides had rendered *P. xylostella* considerably resistant, while negatively affecting the survival of its natural enemies. It can be assumed that, in today’s world, where pests like diamondback moths have developed a strong resistance to organophosphate insecticides, continuing the excessive use of such insecticides will be extremely detrimental to their natural predator insects (e.g., *Cotesia plutellae*, *Pteromalus puparum*). The I50 values of the two OPs against *P. striolata* were only 1/6~1/18 of those against *P. xylostella*, indicating that the field resistance of *P. striolata* is not as strong as that of lepidopteran *P. xylostella*. This may be because the larval stage of *P. striolata* mainly grows underground, which results in a lower contact with the pesticide compared to *P. xylostella*, which feeds on leaves, thus slowing down the development of resistance. The AChE of *M. domestica* and *C. porcellus*, which were not subjected to external insecticide pressure, exhibited greater I50 values than those of *P. striolata*, *P. japonica*, *P. litura*, etc., which were subjected to insecticide selection pressure under natural conditions. This phenomenon might be due to different degrees of species evolution, or due to species-specific differences among organisms with similar degrees of evolution. Furthermore, the AChE I50 of the corresponding organisms inhibited by the two OPs showed significant differences (Table 1), indicating that the OP type affected the target biological AChE sensitivity (*t*-test, *p* ≤ 0.05, different uppercase or lowercase letters indicate significant differences between the two). Overall, the toxicity of paraoxon and malaoxon on mammals is similar to previous reports [25]. However, there are almost no reports on different insects.

### 2.2. The Spontaneous Reactivation Rate Constants (Ks) of AChE Inhibited by Paraoxon and Malaoxon

**Figure 2:**
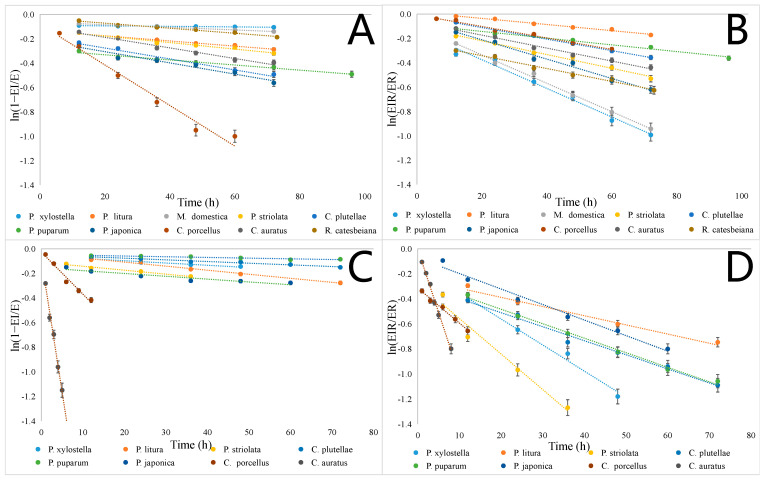
We used I90 concentrations of paraoxon and malaoxon to inhibit ten AChE. (**A**) The spontaneous reactivation of ten AChE inhibited by paraoxon. (**B**) The aging of ten AChE inhibited by paraoxon. (**C**) The spontaneous reactivation of eight AChE inhibited by malaoxon. (**D**) The aging of eight AChE inhibited by malaoxon. The error bars were calculated as percentages. In graphs (**A**,**C**), the slope of the curve represents the Ks of the AChE. In graph (**B**,**D**), the slope of the curve represents the Ka of the AChE. (The data were from three replicates, and are presented as the mean, n = 3). Additionally, please refer to Table 3 for specific statistical values (the regression equation and R2 of regression curve).

After the toxicity test, the spontaneous reactivation test aimed to explore the recovery ability of the target AChE. We used the I90 concentration OPs to maintain the enzyme activity of the AChE of ten species at the same level. Importantly, in the pre-experiment, we tested the reaction rates of paraoxon and malaoxon inhibiting the AChE of ten species. This can allow us to estimate the time range and interval of testing in AChE recovery testing (Table 2). To accurately compare the differences in the Ks values of ten AChE inhibited by OPs, relevant calculations (Table 3) were conducted based on equation ln(1 −EI/E) = −Kst, and Figure 2 was drawn. It was obvious that, in the paraoxon test, the activity of *C. porcellus* AChE changed very quickly compared with the other nine AChE (Figure 2). In addition, in the maraoxon test, the most prominent species AChE were those of *C. porcellus* and *C. auratus*. Compared to other AChE, the recovery ability was relatively high. 

According to Table 4, the spontaneous reactivation rate constants of the same drug differed among species. Although some differences in the AChE reactivation constant order were noted between paraoxon and malaoxon, *C. auratus* and *C. porcellus* showed a strong AChE recovery ability after inhibition by the two OPs. The AChE of *P. striolata* and *P. japonica* had a high Ks after inhibition by the two OPs, indicating that the dephosphorylation ability of Coleoptera after inhibition by OPs might be stronger than that of other insect groups. Furthermore, the AChE of *P. xylostella* and *P. litura* inhibited by paraoxon showed a small Ks, indicating that the dephosphorylation ability of Lepidoptera after paraoxon poisoning was very weak. The *C. porcellus* AChE Ks values after inhibition by the two OPs were very large, and exceeded those of all the insects, indicating that the recovery ability of mammalian AChE inhibited by OPs was much stronger than that in insects. Importantly, the *P. xylostella* trapped with some resistance in the field possessed a higher degree of insensitivity to OP than indoor-reared *M. domestica*. The Ks of *P. xylostella* was 0.02, while that of *M. domestica* was 0.11, and the recovery ability of the *M. domestica* AChE was much higher than that of *P. xylostella*, further demonstrating that the insect Ks was mainly affected by species differences, while the relationship with the organisms’ resistance to OP was not apparent. Similarly, although *P. xylostella* was much less sensitive to OP than its natural enemies, *C. plutellae* and *P. japonica*, the Ks values of the latter were 22 greater than that of *P. xylostella*, reconfirming the above conclusion. A comprehensive analysis of these Ks values showed some significant correlations with the OP type and the enzyme source (*t*-test, *p* ≤ 0.05).

### 2.3. The Aging Rate Constant (Ka) of AChE Inhibited by Paraoxon and Malaoxon

Interestingly, in the aging constant test, the aging rate of *C. porcellus* and *C. auratus* AChE-inhibited malaoxon was relatively high. The enzyme activity data required for the test can be obtained within 12 h (Table 3). In addition, based on the equation ln(EIR/ER) = −Kat, and the activity data measured in ten different species, we calculated the value of Ka (Table 5), and created Figure 2. As shown in Table 5, the order of the Ka of the AChE inhibited by paraoxon was *P. xylostella* > *M. domestica* > *P. japonica* > *C. auratus* > *P. striolata* > *R. catesbeiana* > *C. plutellae* = *C. porcellus* > *P. puparum* > *P. litura*. The order of the Ka of the AChE inhibited by malaoxon was *C. auratus* > *P. striolata* > *C. porcellus* > *P. xylostella* > *P. japonica* > *P. puparum* > *C. plutellae* > *P. litura*. Ka represents the complete inactivation of phospho-AChE. Once phospho-AChE ages, it cannot be reactivated by known activators. Based on this, with a smaller Ka, it is beneficial to the phospho-AChE to have more time to spontaneously dephosphorylate, promoting drug resistance. Interestingly, *C. auratus* had a low susceptibility to Ops, but showed a high Ka after being suppressed by the OPs, particularly by malaoxon (Ka = 10.59). The same phenomenon was also found when the AChE of *C. porcellus* and *R. catesbeiana* were inhibited by the OPs. Moreover, the susceptibility of *P. xylostella* to the OPs was lower than that of *P. japonica*, *C. plutellae*, *P. puparum*, and *P. litura*, but its Ka was larger than that of other insects, indicating that the Ka value was species-specific. The similar Ka values of the indoor-raised *M. domestica* and *P. xylostella* reconfirm the above. The AChE Ka values in *C. porcellus* and *C. auratus* after inhibition by malaoxon were smaller than their Ks values, proving that the AChE in evolutionarily advanced organisms might have a better self-recovery ability. It was also noted that the Ka of AChE inhibited by malaoxon was larger than that of the corresponding materials inhibited by paraoxon, indicating a possible close correlation between the OP type and the Ka of phospho-AChE. Further comparison of the Ka and Ks values of all the tested insects showed a tendency for greater Ka than Ks values following OP exposure. Moreover, the AChE activity of *M. domestica* inhibited by malaoxon did not recover naturally, nor did it recover after adding various concentrations of several activators; therefore, its aging constant could not be calculated. When malaoxon inhibited the AChE of *R. catesbeiana*, the reactivation rate after the addition of the activator was too slow to obtain a value that could be used for the calculations, so this Ka was not obtained.

## 3. Discussion

Paraoxon, a metabolite of parathion, inhibits AChE in higher animals more than parathion. Similarly, malaoxon, a malathion metabolite, inhibits AChE more effectively than malathion. Their mode of action is of considerable significance in diethoxy phosphate insecticides [26,27,28]. The malaoxon toxicity to AChE in the ten species assessed varied much less than the toxicity of paraoxon. In addition, it was not difficult to see that the AChE of organisms with better evolutionary levels, such as *R. catesbeiana*, *C. auratus*, and *C. porcellus*, under the inhibition of OP, had better insensitivity to two types of OP pesticide. As shown in Table 1 and Table 2, it was intuitively obvious that the AChE of *C. porcellus* was very sensitive to paraoxon, but not as sensitive to malaoxon. These results suggested that malaoxon was not as toxic as paraoxon to higher animals, but had a better selectivity than paraoxon. This characteristic contributes to pesticide safety in higher animals. While parathion insecticides have been banned, malathion is still widely used, and is a valuable alternative to replace pesticides with a high toxicity. Moreover, the related toxicological data indicated that parathion was highly toxic to higher animals, while malathion was far less toxic to them [29]. It is generally believed that malathion is strongly selective, as the carboxylesterase of mammals hydrolyzes it much more efficiently than that of insects. In this study, the I50 of *C. porcellus* showed that its AChE was sensitive to paraoxon, but not malaoxon. This may also be closely related to the very low sensitivity of AChE to malathion in higher animals. The IC50 values of paraoxon and malaoxon against the AChE of *P. xylostella* were many times greater than those of its natural enemies, such as *C. plutellae*, *P. puparum*, and *P. japonica*. This study showed that the AChE of these natural enemies were much more sensitive to OPs than that of *P. xylostella*, possibly because these insects rarely feed directly on food containing high pesticide concentrations.

As a metabolic process, the dephosphorylation stage is essential for organisms to defend against covalent binding to the serine of AChE, and the formation of an OP-AChE complex that disrupts the catalytic triad. This defense mechanism can promote the development of resistance in various organisms [30,31]. The dissociation of the OP complex is mainly due to the nucleophilic attack of a water molecule on the P atom [32,33]. The Ks of the ten AChE types inhibited by the two OPs showed that *C. porcellus* included significantly higher Ks than other species. The strong detoxification capability of higher animals was closely related to the efficient dephosphorylation reaction that attenuated the effect of the two OPs. Our previous studies reported that the differences in the Ki values of AChE exposed to OPs in pests and their natural enemies were related to their susceptibility to insecticides (LC50). The pest AChE were inhibited by Ops, and had higher IC50 and lower ki values than those of beneficial insects. Following this in-depth study of various organisms, the Ka and Ks revealed that the recovery and aging responses of their AChE following suppression by OPs were mostly species-specific, rather than related to differences in resistance to OPs between the pests and their natural enemies. Additionally, compared to other insects in this study, Coleoptera insects demonstrated a superior dephosphorylation ability following AChE inhibition. Aging has a smaller energy barrier than dephosphorylation, resulting in the formation of reactivation-resistant OP–AChE complexes that involve Gly122, an essential residue for the dealkylation (aging) and dephosphorylation (reactivation) of OP–AChE [15]. Our studies found that the Ka values of malaoxon were generally greater than those of paraoxon, proving that the value of Ka highly depended on the insecticide type. When the Ka and Ks of the insects were compared, the Ka values were generally greater than the Ks values. This might be because OPs are highly toxic to insects, causing an almost irreversible toxicity, and showing no selectivity in the pest populations. 

In addition, the highlights of this experiment can be found in the Supplementary Materials.

## 4. Materials and Methods

### 4.1. Sources of Biological Materials

*Plutella xylostella*: harvested from the wild and raised to the first generation (F1); *Propylea japonica*, *Pteromalus puparum*, and *Prodenia litura*: raised in vegetable plots (the F1 generation obtained via breeding); *Phyllotreta striolata*: captured at Shangjie Town, Minhou (the F1 generation obtained via breeding); *Cotesia plutellae*: the *P. xylostella* larvae were captured in the vegetable fields of Shangjie Town, Minhou. Their parasites formed white bee pupae that feathered into adult *C. plutellae*; *Musca domestica*, and *Cavia porcellus* were donated by the Fujian Provincial Center for Disease Control and Prevention (sensitive to OPs; neither their parents nor themselves have been exposed to organophosphorus pesticides). For OP poisoning, the guinea pig (*Cavia porcellus*) is a commonly used animal model because guinea pigs more closely mirror primate susceptibility to OP poisoning than other animals do, such as rats or mice. *Carassius auratus* and *Rana catesbeiana* were purchased at Fuzhou Yonghui Supermarket.

### 4.2. Chemicals

The following chemicals were purchased from Sigma( Beijing, China ): paraoxon (Diethyl 4-nitrophenyl phosphate; ≥95%); malaoxon (Diethyl 2-(dimethoxyphosphorylsulfanyl)butanedioate; ≥95%); acetylthiocholine iodide (ATCh); 5,5′-dithiobis-(2-nitrobenzoic acid) (DTNB); Triton. We also used trimedoxime (TMB4; Fluka, shanghai, China) and reversed-phase silica (500 mg/3 mL, Bakerbond, Beijing, China). High speed freezing centrifuge ( Dalong Xingchuang Experimental Instrument Co., Ltd., Beijing, China).Although the use of these two organophosphorus pesticides has been banned in some regions, paraoxon and malaoxon are still highly representative organophosphorus pesticides in the study of acetylcholinesterase poisoning.

### 4.3. Preparation of AChE

AChE preparation followed a previously described procedure, with some modifications [34]. The *C. porcellus* were killed via cervical dislocation; the *C. auratus* and *R. catesbeiana* were decapitated. Other species were ground with liquid nitrogen. Based on our previous and current biological analysis, the treatment methods for the ten biomaterials used in the study are presented in Table 6 [20]. It should be noted that the supernatants would be interfered with by suspended fat if the whole bodies of *P. xylostella* fourth instar larvae and adults of the ladybugs *M. domestica* and *P. litura* were used for AChE enzyme preparation, even after filtering the homogenates through glass wool. The prepared biological materials were immediately added to PBS buffer (pH = 7.4, concentration 0.067 mol·L^−1^) supplemented with 0.1% (*v*/*v*) Triton X-100 ( Adamas life, Shanghai, China ), 0.003 mol·L^−1^ EDTA ( Adamas life, Shanghai, China ), and phenylthiourea at certain concentrations, pre-cooled in a 4 °C ice bath, and homogenized via a tissue homogenizer. The above facilitated the separation and extraction of the AChE, and effectively prevented enzyme oxidation and chelation by heavy metals, thereby ensuring complete enzyme activity. After the mixture was centrifuged at 10,000 rpm for 15 min at 4 °C, the supernatants were used to measure the enzymatic activity of the AChE. Three animal samples were prepared for each assay. The protein concentrations in the enzyme preparations were about 5.0 mg·mL^−1^ in all the biological samples. The protein concentrations were determined via the Bradford method, using bovine serum albumin as a standard.

### 4.4. Determination of AChE Activity

The AChE activity was determined following the Ellman measurement method described by Wu and Miyata [20]. ATCh, used in experiments as a substrate for AChE, can be decomposed into thiocholine and acetic acid. Thiocholine can react with the chromogenic agent DTNB to generate a yellow substance that absorbs light at 412 nm. The reaction system consisted of 2.0 mL PBS (pH = 7.4, concentration 0.067 mol·L^−1^, Adamas life, Shanghai, China), 50 µL of 0.08 mol·L^−1^ ATCh, and 50 µL of 0.02 mol·L^−1^ DTNB. Enzyme solution (50–100 µL) was added to the reaction system, depending on the species-specific enzyme activity. After incubation for 20 min at 25 °C, the optical density of the generated yellow product was measured at a wavelength of 412 nm. 

### 4.5. Determination of the Sensitivity of AChE Inhibited by Paraoxon and Malaoxon

Paraoxon and malaoxon were separately diluted into a series of concentration solutions, using acetone. Then, the enzyme solution and the drug were evenly mixed, in a ratio of 400:25. After being kept at 25 °C for 30 min, 50–100 µL of the mixed solution was taken based on the species-specific AChE activity, and the AChE activity was measured as poisoned enzyme activity (EI). Pure acetone solution was used as a control, to measure normal enzyme activity (E). The degree of enzyme inhibition was calculated from the activities of the inhibited and control samples. The original drug was diluted into 5–6 concentrations (0.125–3200 ppm; there were differences in the sensitivity to OP among different AChE) to inhibit the AChE, and the inhibitory rate was calculated at the various concentrations. Each experiment was repeated three times. Following Probit analysis with SPSS 13.0, LD-P was drawn, and the regression equation was tested using the X2 formula. Finally, the medium (I50) and the 90% (I90) inhibition concentrations of AChE by paraoxon and malaoxon were calculated [34].

Rate of inhibition (%) = (E − EI)/E × 100%

### 4.6. Spontaneous Reactivation Rate Constants (Ks) of AChE Inhibited by Paraoxon and Malaoxon

Before the spontaneous reactivation test, we conducted a pre-experiment, to test the reaction rates of different AChE with two different OPs. Then, the time range and interval for measuring the enzymatic activity of different AChE can be estimated. We inhibited the enzymes by preincubating the tissue homogenates with the I90 concentration of paraoxon or malaoxon for 30 min at 25 °C. This can inhibit enzyme samples at the same level. Following inhibition, excess pesticides were removed using a C18 reverse phase column (Bakerbond, C18; 3 × 50 mg·mL^−1^). The extracted enzyme solution was stored at 25 °C in a water bath [35]. The same volume of acetone was used, and the same procedure was followed, for the control experiment. The enzyme activity in the solution was measured at fixed intervals (in general, the enzyme activity was measured six times with a 12 h interval. However, the specific procedures were adjusted accordingly, based on the characteristics of different biological enzymes). The experiments were performed in triplicate, to obtain the most accurate results. The graph was plotted using the following equation, to obtain the reactivation constant Ks [25], represented by the slope:

ln(1 − EI/E) = −Kst

EI, the enzyme solution activity after inhibition; E, the control enzyme solution activity;Ks, spontaneous reactivation constant, t, the time in hours since inhibition.

### 4.7. Aging Rate Constants (Ka) of AChE Inhibited by Paraoxon and Malaoxon

The test method referred to previous reports, and made appropriate improvements [35]. The AChE was inhibited with the I90 drug concentration at 25 °C for 30 min. Subsequently, the remaining pesticides were removed through a C18 reverse-phase column, and stored at 25 °C. Pure acetone solvent was used as the control. After removing residual pesticides, 300 µL of enzyme solution was extracted at regular intervals, and 20 µL of TMB-4 (1.3 mmol·L^−1^, 37 °C) was added, to reactivate the enzyme solution, maintained at 25 °C. After incubation in a water bath for about 15 min, the enzyme activity was measured. In general, the enzyme activity following reactivation was measured at least six times, with 12 h intervals between measurements, and the relevant data were calculated. The aging rate constant (Ka) was calculated from the slope of the following equation, using the least-squares method. Each assay was repeated three times.

ln(EIR/ER) = −Kat

ER is the control enzyme activity after reactivation; EIR is the inhibited enzyme activity after reactivation; Ka represents the aging constant and t is the time after inhibition in hours.

### 4.8. Data Analysis

The experimental data were recorded and assessed in Excel 2013 (16.0.16731.20052, Microsoft, Washington, DC, USA). SPSS 13.0 (28.0.1.1, SPSS Inc., Chicago, IL, USA) was used for further data calculations, and to ensure that the data were accurate and reliable.

## 5. Conclusions

Overall, our experiment selected two representative OPs, and ten biological materials. The characteristics of the AChE were compared between insect species, Coleoptera, Lepidoptera, and Diptera, pests and their natural enemies, and insects and vertebrates, and accurate data were obtained. As no similar report has been published, our study provides a reliable basis for further research on the mechanism of pest resistance, species evolution, the relevant parameters of AChE, and the mechanism of action of OPs.

## Figures and Tables

**Table 1:** The sensitivity of AChE inhibition by paraoxon and malaoxon.

**Species**		**Paraoxon**	
	**Toxicity Regression Equation**	**I_50_ (mol·L** **^−^** **^1^** **)**	**I_90_ (mol** **·** **L** **^−^** **^1^** **)**
*Plutella xylostella* (moth)	y = 1.02x + 3.57	(5.88 ± 0.434) × 10^−6^ A	(1.297 ± 0.446) × 10^−4^
*Prodenia litura* (cotton leafworm)	y = 1.25x + 4.61	(4.46 ± 0.516) × 10^−7^ B	(5.234 ± 0.592) × 10^−6^
*Musca domestica* (housefly)	y = 0.87x + 3.94	(3.17 ± 0.378) × 10^−6^ C	(1.127 ± 0.064) × 10^−4^
*Phyllotreta striolata* (flea beetle)	y = 1.10x + 4.83	(3.27 ± 0.220) × 10^−7^ D	(4.811 ± 0.285) × 10^−6^
*Cotesia plutellae* (wasp)	y = 2.49x + 6.19	(7.21 ± 0.064) × 10^−8^ E	(2.334 ± 0.004) × 10^−7^
*Pteromalus puparum* (parasitic wasp)	y = 2.20x + 5.96	(7.77 ± 0.128) × 10^−8^ F	(2.973 ± 0.047) × 10^−7^
*Propylea japonica* (ladybird)	y = 2.11x + 5.79	(9.85 ± 0.086) × 10^−8^ G	(4.467 ± 0.077) × 10^−7^
*Cavia porcellus* (guinea pig)	y = 3.02x + 6.17	(8.40 ± 0.300) × 10^−8^ H	(2.308 ± 0.054) × 10^−7^
*Carassius auratus* (goldfish)	y = 2.45x + 1.18	(7.50 ± 0.201) × 10^−6^ I	(2.698 ± 0.112) × 10^−5^
*Rana catesbeiana* (frog)	y = 2.31x – 1.71	(1.74 ± 0.006) × 10^−4^ J	(6.130 ± 0.144) × 10^−4^
**Species**		**Malaoxon**	
	**Toxicity Regression Equation**	**I_50_ (mol** **·** **L** **^−^** **^1^** **)**	**I_90_ (mol** **·** **L** **^−^** **^1^** **)**
*Plutella xylostella*	y = 1.80x + 1.83	(1.06 ± 0.022) × 10^−5^ A *	(4.34 ± 0.146) × 10^−5^
*Prodenia litura*	y = 1.50x + 3.68	(1.40 ± 0.026) × 10^−6^ B *	(9.38 ± 0.671) × 10^−6^
*Musca domestica*	y = 1.53x + 3.22	(2.74 ± 0.036) × 10^−6^ C *	(1.92 ± 0.060) × 10^−5^
*Phyllotreta striolata*	y = 0.84x + 4.18	(1.73 ± 0.016) × 10^−6^ B *	(2.76 ± 0.443) × 10^−5^
*Cotesia plutellae*	y = 1.85x + 4.36	(5.21 ± 0.406) × 10^−7^ D *	(4.30 ± 0.656) × 10^−6^
*Pteromalus puparum*	y = 2.35x + 4.26	(3.88 ± 0.055) × 10^−7^ E *	(1.39 ± 0.038) × 10^−6^
*Propylea japonica*	y = 1.84x + 3.72	(9.42 ± 0.321) × 10^−7^ F *	(3.49 ± 0.077) × 10^−6^
*Cavia porcellus*	y = 1.63x + 2.33	(7.74 ± 0.300) × 10^−6^ G *	(4.72 ± 0.278) × 10^−5^
*Carassius auratus*	y = 1.23x + 2.88	(1.02 ± 0.040) × 10^−5^ A *	(1.10 ± 0.056) × 10^−4^
*Rana catesbeiana*	y = 2.24x – 0.24	(3.95 ± 0.097) × 10^−5^ H *	(1.54 ± 0.062) × 10^−4^

The effects of AChE inhibition by paraoxon and malaoxon in ten species were calculated using various OP concentrations. The toxicity on AChE can be compared using I_50_, and paraoxon and malaoxon with I_90_ concentration were used for the aging and recovery testing of AChE. Different uppercase letters in the same column indicate significant differences in the I_50_ of various species inhibited by the same OP (*t*-test, *p* ≤ 0.05). * indicates that the AChE I_50_ of the same species differs significantly between OPs (*t*-test, *p* ≤ 0.05). The data were obtained from three replicates, and presented as the mean.

**Table 2:** The test time and OP concentration in aging and recovery tests.

Species		Paraoxon		Malaoxon
	Test Time (h)	Test Concentration (mol·L^−1^)	Test Time (h)	Test Concentration (mol·L^−1^)
P.X.	72	(1.297 ± 0.446) × 10^−4^	48	(4.34 ± 0.146) × 10^−5^
P.L.	72	(5.234 ± 0.592) × 10^−6^	72	(9.38 ± 0.671) × 10^−6^
M.D.	72	(1.127 ± 0.064) × 10^−4^	-	(1.92 ± 0.060) × 10^−5^
P.S.	72	(4.811 ± 0.285) × 10^−6^	36	(2.76 ± 0.443) × 10^−5^
C.L.	72	(2.334 ± 0.004) × 10^−7^	72	(4.30 ± 0.656) × 10^−6^
P.P.	96	(2.973 ± 0.047) × 10^−7^	72	(1.39 ± 0.038) × 10^−6^
P.J.	72	(4.467 ± 0.077) × 10^−7^	60	(3.49 ± 0.077) × 10^−6^
C.P.	60	(2.308 ± 0.054) × 10^−7^	12	(4.72 ± 0.278) × 10^−5^
C.A.	72	(2.698 ± 0.112) × 10^−5^	8	(1.10 ± 0.056) × 10^−4^
R.C.	73	(6.130 ± 0.144) × 10^−4^	-	(1.54 ± 0.062) × 10^−4^

The I_90_ concentration OPs were used to maintain the enzyme activity of ten AChE at the same level. Abbreviations: P.X., *Plutella xylostella*; P.S., *Phyllotreta striolata*; P.J., *Propylea japonica*; C.L., *Cotesia plutellae*; C.P., *Cavia porcellus*; P.P., *Pteromalus puparum*; C.A., *Carassius auratus*; M.D., *Musca domestica*; P.L., *Prodenia litura*; and R.C., *Rana catesbeiana*.

**Table 3:** The statistical data for Figure 2.

Species	Paraoxon		Malaoxon	
	Reactivation (Figure 2A)	Aging (Figure 2B)	Reactivation (Figure 2C)	Aging (Figure 2D)
*P. xylostella*	y = −0.0002x − 0.0896 R^2^ = 0.9516	y = −0.0118x − 0.1391 R^2^ = 0.9821	y = −0.0018x − 0.0595 R^2^ = 0.9605	y = −0.0208x − 0.1452 R^2^ = 0.9852
*P. litura*	y = −0.0021x − 0.1324 R^2^ = 0.9758	y = −0.0025x + 0.0155 R^2^ = 0.9865	y = −0.0033x − 0.042 R^2^ = 0.984	y = −0.0073x − 0.2411 R^2^ = 0.9709
*M. domestica*	y = −0.0011x − 0.0634 R^2^ = 0.9932	y = −0.0117x − 0.1019 R^2^ = 0.9955	-	-
*P. striolata*	y = −0.0027x − 0.1249 R^2^ = 0.9743	y = −0.0052x − 0.1055 R^2^ = 0.9853	y = −0.0028x − 0.1092 R^2^ = 0.9775	y = −0.0284x − 0.274 R^2^ = 0.9656
*C. plutellae*	y = −0.0045x − 0.186 R^2^ = 0.975	y = −0.0047x − 0.0154 R^2^ = 0.9946	y = −0.0014x − 0.0488 R^2^ = 0.9743	y = −0.0112x − 0.2867 R^2^ = 0.9859
*P. puparum*	y = −0.0021x − 0.2893 R^2^ = 0.9768	y = −0.0028x − 0.0866 R^2^ = 0.9874	y = −0.0006x − 0.0468 R^2^ = 0.9012	y = −0.0116x − 0.2516 R^2^ = 0.9939
*P. japonica*	y = −0.0046x − 0.2135 R^2^ = 0.9607	y = −0.0079x − 0.0518 R^2^ = 0.9835	y = −0.0023x − 0.1542 R^2^ = 0.9042	y = −0.0131x − 0.0735 R^2^ = 0.9824
*C. porcellus*	y = −0.0171x − 0.0601 R^2^ = 0.9589	y = −0.0047x − 0.0064 R^2^ = 0.9831	y = −0.0348x − 0.0262 R^2^ = 0.9774	y = −0.0281x − 0.3133 R^2^ = 0.9891
*C. auratus*	y = −0.0043x − 0.1013 R^2^ = 0.9776	y = −0.0053x − 0.0658 R^2^ = 0.9862	y = −0.2173x − 0.0816 R^2^ = 0.9845	y = −0.1061x + 0.012 R^2^ = 0.9826
*R. catesbeiana*	y = −0.0021x − 0.0308 R^2^ = 0.9832	y = −0.0053x − 0.2359 R^2^ = 0.9857	-	-

Table 3 displays the specific statistical data for Figure 2.

**Table 4:** The spontaneous reactivation rate constants of AChE inhibited by paraoxon and malaoxon.

Species	Paraoxon (Ks, %/h)	Malaoxon (Ks, %/h)
*Plutella xylostella*	0.02 ± 0.01 A	0.18 ± 0.01 A *
*Prodenia litura*	0.20 ± 0.01 B	0.32 ± 0.02 B *
*Musca domestica*	0.11 ± 0.01 C	-
*Phyllotreta striolata*	0.27 ± 0.02 D	0.27 ± 0.05 BD
*Cotesia plutellae*	0.45 ± 0.02 E	0.14 ± 0.03 A *
*Pteromalus puparum*	0.21 ± 0.02 B	0.05 ± 0.04 C *
*Propylea japonica*	0.45 ± 0.01 E	0.23 ± 0.03 D *
*Cavia porcellus*	1.71 ± 0.03 F	3.50 ± 0.10 E *
*Carassius auratus*	0.44 ± 0.02 E	21.71 ± 0.81 F *
*Rana catesbeiana*	0.22 ± 0.01 B	-

Different uppercase letters in the same column indicate significant differences among the AChE Ks values of the various species inhibited by the same OP (*t*-test, *p* ≤ 0.05). * indicates that the AChE Ks values of the same organism differ significantly between OPs (*t*-test, *p* ≤ 0.05). The data were taken from three replicates, and are presented as the mean ± S.D.

**Table 5:** The aging rate constants of the AChE inhibited by paraoxon and malaoxon.

Species	Paraoxon (Ka, %/h)	Malaoxon (Ka, %/h)
*Plutella xylostella*	1.18 ± 0.06 A	2.02 ± 0.07 A *
*Prodenia litura*	0.25 ± 0.02 B	0.73 ± 0.01 B *
*Musca domestica*	1.17 ± 0.03 A	-
*Phyllotreta striolata*	0.52 ± 0.04 C	2.84 ± 0.29 C *
*Cotesia plutellae*	0.47 ± 0.03 C	1.12 ± 0.07 D *
*Pteromalus puparum*	0.28 ± 0.02 D	1.15 ± 0.03 D *
*Propylea japonica*	0.78 ± 0.07 E	1.30 ± 0.06 E *
*Cavia porcellus*	0.47 ± 0.02 C	2.81 ± 0.11 C *
*Carassius auratus*	0.53 ± 0.04 C	10.59 ± 1.06 F *
*Rana catesbeiana*	0.48 ± 0.05 C	-

Different uppercase letters in the same column indicate significant differences in Ka among organisms inhibited by the same OP (*t*-test, *p* ≤ 0.05). * indicates that the Ka of the AChE from the same organism differed significantly between OPs (*t*-test, *p* ≤ 0.05). The data were taken from three replicates, and are presented as the mean ± S.D.

**Table 6:** The treatments of the biological materials.

Species	Processing Method
*Plutella xylostella* (moth)	After eclosion, complete *P. xylostella* adults were raised for over 24 h using 10% honey water, before being frozen in liquid nitrogen and homogenized with a homogenizer. Approximately 4–5 adults were required per milliliter of homogenate.
*Prodenia litura* (cotton leafworm)	Their heads and chests were homogenized, after the freezing of complete adults in liquid nitrogen. Approximately two adult heads and chests were required for each milliliter of uniform slurry.
*Musca domestica* (housefly)	Raised with honey water until after *M. domestica* eclosion, complete adults were placed in liquid nitrogen, and their heads were homogenized. Approximately two *M. domestica* were required per milliliter of homogenate.
*Phyllotreta striolata* (flea beetle)	Complete adults were quickly homogenized, after treatment with liquid nitrogen. Approximately 20 adults were required per milliliter of homogenized liquid.
*Cotesia plutellae* (wasp)	Complete adults after eclosion were treated with liquid nitrogen, and then quickly homogenized. Approximately five adults were required per milliliter of homogenized liquid.
*Pteromalus puparum* (parasitic wasp)	After eclosion, *P. puparums*, parasitized within the puparium, were treated with liquid nitrogen, and quickly homogenized. Approximately 6–8 mL of homogenate can be prepared from the adult *P. puparums* produced by one bee pupa.
*Propylea japonica* (ladybird)	The head and chest were homogenized after the treatment of complete adults with liquid nitrogen. Approximately five adults were required for each milliliter of homogenate.
*Cavia porcellus* (guinea pig)	The brain of *C. porcellus* was homogenized at a ratio of 1:20 (*w*/*v*).
*Carassius auratus* (goldfish)	The brain of *C. auratus* was homogenized at a ratio of 1:20 (*w*/*v*).
*Rana catesbeiana* (frog)	The brain of *R. catesbeiana* was homogenized at a ratio of 1:20 (*w*/*v*).

## Data Availability

All data generated or analyzed during this study are included in this article, and materials are available from the authors upon reasonable request.

## Supplementary Materials

The following supporting information can be downloaded at: https://www.mdpi.com/article/10.3390/ijms241814213/s1.
