# Supplementary material for: Study on Spontaneous Reactivation and Aging of Acetylcholinesterase Inhibited by Paraoxon and Malaoxon in Ten Species
Source: Int J Mol Sci. 2023 Sep 18;24(18):14213. doi: 10.3390/ijms241814213 (PMC10531824; doi:10.3390/ijms241814213)
Supplement: Supplementary file 1 [file ijms-24-14213-s001.zip › ijms-2600611-supplementary.pdf]

Highlights

- The Ka and Ks of AChE inhibited by OP showed mainly species-specific correlations and little correlation with sensitivity to OP.
- Varieties of organic phosphorus pesticides strongly affected the aging constant of Phospho-AChE.
- AChE, from ten different organisms, was much more differentially sensitive to paraoxon than to malaoxon.
- Cavia porcellus AChE was sensitive to paraoxon and poorly sensitive to malaoxon.
- The recovery of phosphorylated AChE was greater in Coleoptera than in other insects.
- When insects developed organophosphorus poisoning, they were more inclined to experience aging reactions.
